# Response to the letter to the editor Fibrosis or steatosis: which is the best screening target? Comment on the Brazilian evidence-based guideline for screening, diagnosis, treatment, and follow-up of metabolic dysfunction-associated steatotic liver disease (MASLD) in adult individuals with overweight or obesity

**DOI:** 10.20945/2359-4292-2024-0093

**Published:** 2024-06-03

**Authors:** Rodrigo O. Moreira

**Affiliations:** 1 Instituto Estadual de Diabetes e Endocrinologia Luiz Capriglione Rio de Janeiro RJ Brasil Instituto Estadual de Diabetes e Endocrinologia Luiz Capriglione, Rio de Janeiro, RJ, Brasil

## DEAR EDITOR AND COLLEAGUES,

The letter by Mateus Severo raises important questions regarding the screening and diagnosis of metabolic dysfunction-associated steatotic liver disease (MASLD) in adult individuals with overweight or obesity. The points presented in the letter have been discussed in detail by the authors during the Brazilian Evidence-Based guideline elaboration (1) and deserves further clarification.

Liver steatosis is the most common manifestation and the hallmark feature of MASLD. It is now considered to be an independent risk factor for cardiovascular diseases (2) and it is also strongly correlated to metabolic disturbances (3). Therefore, it may indicate that overweight and obese individuals with liver steatosis deserves a specific and individualized approach. Although the authors strongly agree that the identification of liver fibrosis is one of the major goals in the evaluation of patients with MASLD, they also reinforce that screening for liver steatosis should not be neglected.

Abdominal Ultrasound does have limitations, particularly in individuals with Body Mass Index (BMI) > 35 kg/m^2^. However, as stated in the letter, "the projected prevalence of overweight and obesity in Brazil for 2030 is 68.1% and 29.6%, respectively (4)". The vast majority of individuals with overweight will have a BMI between 25 and 30 kg/m^2^. In this population, in which the estimated prevalence of liver steatoses may be as low as 40% (5), an exam with a sensitivity of 85% may be more than adequate to identify patients. The meta-analysis by Ruben Hernaez and cols. (5) cited by Mateus Severo concludes "that liver ultrasonography is an accurate, reliable tool to detect moderate to severe fatty liver, with sensitivity and specificity of 84.8% and 93.6%…". Moreover, it also states that "together with the relatively low cost and lack of radiation exposure, support the use of ultrasound as the imaging technique of choice for screening for fatty liver in clinical settings and population studies".

Finally, the panel strongly believes that there is an important gap on the knowledge of liver steatoses by non-specialists. A significant number of physicians needs clarification about what should be and what should not be done in overweight individuals with steatosis. Therefore, the guideline provides evidence-based recommendations for the most common manifestation of MASLD.
